# Cascaded Semantic Fractionation for identifying a domain in social media

**DOI:** 10.3389/frma.2024.1189099

**Published:** 2024-03-01

**Authors:** James Danowski, Ken Riopelle, Bei Yan

**Affiliations:** ^1^Department of Communication, University of Illinois at Chicago, Chicago, IL, United States; ^2^Department of Industrial and Systems Engineering, Wayne State University, Detroit, MI, United States; ^3^School of Business, Stevens Institute of Technology, Hoboken, NJ, United States

**Keywords:** semantic networks, Facebook, CrowdTangle, start words, COVID-19

## Abstract

Searching social media to find relevant semantic domains often results in large text files, many of which are irrelevant due to cross-domain content resulting from word polysemy, abstractness, and degree centrality. Through an iterative pruning process, Cascaded Semantic Fractionation (CSF) systematically removes these cross-domain links. The social network procedure performs community detection in semantic networks, locates the semantic groups containing the terms of interest, excludes intergroup links, and repeats community detection on the pruned intragroup network until the domain of interest is clarified. To illustrate CSF, we analyzed public Facebook posts, using the CrowdTangle app for historical data search, from February 3, 2020, to March 13, 2021, about the possible Wuhan lab leak of COVID-19 over a daily interval. The initial search using keywords located six multi-day bursts of posts of more than 500 per day among 95 K posts. These posts were network analyzed to find the domain of interest using the iterative community detection and pruning process. CSF can be applied to capture the evolutions in semantic domains over time. At the outset, the lab leak theory was presented in conspiracy theory terms. Over time, the conspiratorial elements washed out in favor of an accidental release as the issue moved from social to mainstream media and official government views. CSF identified the relevant social media semantic domain and tracked its changes.

## Introduction

Social media researchers use search terms that capture large files. Much of the content is irrelevant because of the cross-domain linkages of words. One cause is polysemy, the number of different senses a word has. For example, “bank” can mean a financial institution, a river bank, or tilting a plane in a turn. Although English nouns have, on average, 1.23 senses (Jurafsky and Martin, 2018), higher polysemy words draw in content from different domains when they serve as search terms. When the intention is to focus on one domain, cross-domain linkages present a problem.

In addition, some words, although not polysemous, have a higher degree centrality and are more likely network hubs, for example, “love, network, government, health, education, culture, and communication.” Their more significant number of links to other terms increases cross-domain linkages. This is because semantic network hubs tend to be more abstract. Because of their scope, they connect with more domains. A higher degree centrality for seed terms usually affects results more than polysemy because the more abstract words connect more broadly across more diverse domains.

Pruning irrelevant domain content from the search text requires dimensionality reduction from the least structured one-hot state, where every word or pair is a dimension, to some clusters or groups to identify the domain of interest. The main approaches are topic modeling (Vayansky and Kumar, 2020) and community detection (Bedi and Sharma, 2016; Danowski et al., 2021).

Barnett et al. (2023) find that community detection is more effective for representing meaning than topic modeling. Topic modeling suffers from several problems (Boyd-Graber et al., 2014), including the arbitrary setting of k for the number of topics, lack of context because of the bag-of-words approach that ignores word proximity in documents, and interpretability issues.

Cluster analysis, like topic modeling, requires the *a priori* specification of the number of clusters (Milligan and Cooper, 1985). Rather than letting the data generate the cluster solution, the researcher arbitrarily imposes it. Besides the problems of under and over-fitting, there is no fixed parameter to enable comparisons of results. The choice of the distance metric is also an issue. For these reasons, there is no good way to validate cluster analysis results.

In comparison, network-based semantic analysis approaches can be more effective. Community detection, for example, the widely used Clauset et al. (2004) or the Louvain algorithm (Blondel et al., 2008), identifies groups based on a modularity metric that scales to large networks. To start, each node is its community. Then, it merges communities iteratively based on the change in modularity that would result from combining them.

Modularity (Newman, 2006) measures the degree to which a network can be partitioned into non-overlapping communities or modules. It measures the extent to which the links in the network are concentrated within communities rather than between them. The algorithm chooses the pair of communities that would result in the largest increase in modularity, merges them into a single community, and repeats this step until no further increase in modularity is possible.

With such an objective criterion as modularity optimization, community detection identifies groups of words based on a network property, unlike topic modeling and cluster analysis, which specify the number of partitions in advance. As a result, valid comparisons can be made in the network properties associated with semantic groups identified with community detection.

Richards (1974) created a useful visual analogy for finding group network structures. Imagine nodes as balls fixed in place on a table. Attach rubber bands for their links, with thicker bands for stronger ties. Then, release the balls, and the network will configure so that nodes with more shared links will group, and intergroup links will be visible. Groups share most of their connections within the group relative to intergroup links.

Similarly, modularity uses the ratio of within to between community links to find the structure of the network and groups in it. Richards (1974); Vichards (Vichards) and Richards and Rice (1981) created an early form of community detection based on ratios of internal to external group links implemented in NEGOPY.^1^

Nevertheless, while preferable, community detection can produce large groups of words with cross-domain linkages that may complicate interpretation (Fortunato, 2010). The present paper develops an iterative, multi-level form of community detection that removes cross-domain links when locating the domain of interest. We call this process Cascaded Semantic Fractionation (CSF). “Cascaded” means that this process occurs in a series of steps or stages, each building on the previous step's results. “Semantic” refers to the meaning or interpretation of a text, while “fractionation” refers to separating a substance or material into smaller components or fractions. The fractionation process can remove unwanted impurities. For example, distillation is a method of fractionation used to separate a mixture of liquids based on their boiling points.

Consider as a metaphor Russian dolls of decreasing size placed one inside the other, each representing a smaller version of the previous one. Similarly, in Cascaded Semantic Fractionation, a complex semantic structure of nested subdomains is progressively broken down into smaller and smaller components or fractions to reach the desired domain.

The approach is a seed-based semantic network analysis, where derived networks centered on the seed are extracted. An example of seeding a network using another approach is Latent Semantic Scaling (Watanabe and Baturo, 2023). It extracts networks using Latent Semantic Analysis (Deerwester et al., 1990). A key difference is that while Latent Semantic Scaling uses a single network seeding, CSF is an iterative procedure. After the initial seeding, initial word groups are typically large macro-level structures. Large groups of seed terms formed from results are successively pruned based on community identification of word groups. The initial community containing the seed terms is broken down into smaller and smaller subgroups where the nodes in each become the seed terms for the next iteration. This drill-down through layers of the network identifies a micro-level network of relevance.

The starting point in the CSF process is the terms used in the social media search to extract text. For example, in the CrowdTangle database, Boolean searches extract the CSV files of relevant post content. In processing the CSV results file, we select a key term from the search for use in a node-centric extraction of links in the network centered on it. This seed term is often one word or a concatenation of compound terms.

In social network analysis, the most common approach is to examine the one-step links connected with the node. Adding two-step links enables identifying the betweenness centrality of the node in its local network. For example, if the goal were to examine posts about political polarization and e-government and a Boolean search were conducted in CrowdTangle for “(political polarization) AND e-government,” one would likely choose as a seed “e-government” rather than “political polarization” on which to center in the node-centric network analysis. The results would likely show considerably more content for political polarization than e-government. The e-government seed would be better for node-centric analysis because its domain is smaller than that for political polarization. Nevertheless, “political polarization” is likely to appear linked with e-government within two steps in the network.

Neither of these seed-based network representations provides much evidence of meaning because they have limited contextual information, giving only the local network structure of the seed. In contrast, the global group-level context shows how the seed is positioned in a content domain. Because of the network of relationships among words, semantic groups are more likely to suggest meaning narratives than seed-centric networks. The analyst finds a better fit to the narrative that motivated the research, resulting in more understanding and communicability of the results.

A domain is defined more generally as a bounded area of knowledge. Within the boundaries are the relevant concepts and the relationships among these entities. These concepts have stronger within-boundary relationships than external ones. The concepts and relationships define the domain and will manifest as communities of words within semantic networks.

This domain knowledge representation includes a narrative with causal reasoning connecting a group of concepts associated with a seed. Attribution theory has shown that people create causal attributions for what they observe, even when random information is presented (Kelley, 1967, 1973). Individuals need sense-making (Weick, 1995) to create meaning for what they experience. They articulate these meanings through narratives (Brown et al., 2008).

We define a narrative as a statement describing a semantic network's meaning. A narrative may be as short as a sentence or paragraphs long. It is the interpretation of the network structure unit, answering the question, what does the network mean? For example, consider the origin of the seed COVID-19. One narrative centers on the virus originating in another species, a zoonotic transfer to humans through direct contact. Another narrative is based on a leak from the Wuhan Institute of Virology, where the virus was studied.

The analyst typically envisions a narrative that motivates the social media search. Then, when interpreting results, the narrative provides the conceptual reference network for the observed semantic network. As the researcher scans a set of groups from community detection, the narrative provides a criterion for judging which group best fits the domain of interest. After interpreting the results, the narrative may be reinforced or modified.

## Include lists of start words

An essential tool in identifying a seed group is an *include list* of words, the opposite of a stopword list, which can be considered a start-word list (Danowski and Riopelle, 2019). Only the co-occurrences of these terms are identified in producing the network. All other words are dropped. Sources for constructing start words include expert knowledge, special dictionaries, high-frequency words identified in prior research, and names of entities such as countries, organizations, or individuals.

One could use a variety of sources for producing start words. For example, if mapping the semantic networks about an issue were the goal, one might ask subject matter experts to select start words for an include list. Danowski et al. (2023) had three experts select terms from a list of high-frequency words in 54 African countries' policy documents about communication technology development that they thought were most important in evaluating its effects on ICT utilization per capita and included the words that at least two of the experts chose. This include list was then run on each country's documents to profile them and predict ICT utilization per capita as a function of the start words from the policy documents.

Another source for building a list of start words could be prior text-mining research that may have produced particular lexicons for a concept. For example, Loughran and McDonald (2016) analyzed natural business language from annual and quarterly financial reports. One of their lists was for “uncertainty.” Its dictionary contained 275 words they identified from their clustering analysis. Another of their lists is also related to uncertainty, including modal verbs: “may, should, depending, would, and probable.” Danowski and Riopelle (2019) used these terms as an include list of start words to index uncertainty over time in a corpus of news documents associated with the BP Deepwater Horizon Gulf Oil Spill of 2010.

Another approach would be a semantic network analysis to extract key terms in literature about a concept in scholarly journals and books. For example, Cantoni and Danowski (2015) used the start-word approach in editing a book and writing the introductory chapter. First, the other chapters were network analyzed, and the words were examined to select those suitable for an index of the text for the book's end matter. These were fed to an auto-indexing program that produced the book's index terms and page locations. Next, the index terms also formed an include list, which was used in the network analysis of the chapters. Graphs of the network and interpretations were the basis for the introductory chapter to orient the reader to the concepts in the book.

Another use of an include list of start words is for analyzing networks of entities such as countries, organizations, or individuals in news stories. For example, Danowski (2010) and Danowski and Cepela (2010a) used an include list of cabinet member's names to automatically map the social networks of the administrations of presidents, Reagan through G.W. Bush from the member co-mentions in the *New York Times* and *Washington Post*. Danowski (2012c) demonstrated using an include list of publics to examine change over time in Facebook's network of publics over 12 months. Danowski and Riopelle (2019) showed how to build scales for constructs using lists of relevant terms for environmental uncertainty, innovation, strategic planning, and changes in organizational structure.

As Danowski et al. (2021) illustrated, sentiment analysis can be done based on network features. The approach developed a network-based measure of sentiment concerning a target (the name of a person, organization, group, brand, etc.) by identifying the shortest paths connecting the target with sentiment words. To evaluate the semantic network method, they compared the network-based sentiment scores to ground-truth data, sentiment judgments made by human annotators, to see whether the network-based sentiment scores for texts they classified as positive or negative had the expected higher sentiment valence concerning a target. The results validated the semantic network sentiment method. Hegel (2002) developed a similar method, Latent Semantic Scaling, based on principal components analysis of words across documents.

## Semantic network analysis

Semantic network analysis in communication research (Doerfel, 1998; Segev, 2021) can be traced to an analysis of word cooccurrences across posts on the first social media, Computer Bulletin Boards Systems (CBBS), when the first servers based on PCs and modems emerged in the 1980's (Danowski, 1982). The network analysis of words was automated and applied to the email in an organization that observed a crisis during 18 months of study (Danowski and Edison-Swift, 1985), to open-ended survey responses (Rice and Danowski, 1993), and news texts (Danowski, 1993a,b).

The WORDij software package (https//:wordij.net; Danowski, 2013) incorporates this semantic network analysis approach. The critical concept is identifying word pairs by sliding a window, several word positions wide, through the text to code co-occurrences, enabling the network analysis of words (Danowski, 1993a). WORDij parameters enable setting the sliding window width to a desired number of words surrounding each successive word, the radius of the window.

In most research focused on word co-occurrences, bigrams are adjacent words in the text. This reflects a deterministic structure in the network such that the positions of words have a fixed relationship based on the adjacent word bigrams. This approach is highly syntactical. This contrasts with a stochastic approach that incorporates the probabilities of links among words based on their local contexts. The resulting bigrams reveal words that co-occur with less grammatical constraint.

For example, with a sliding window approach, a radius of 1 would produce co-occurrences for adjacent words, a window two words wide. In contrast, a radius of two captures the co-occurrences between words appearing two words before and two words after each word on which the window centers as it slides through the text, with a window width of five. A radius of three captures the co-occurrences among the words appearing three words before and three after the focal word, with a window width of seven words.

Extensive testing (Danowski, 1993b) examined a radius of 1 through 20. It used the network structure of the word co-occurrences, in terms of groups of words, “communities,” in subsequent literature, as a criterion. A radius of three through 20 produced the same word-group network structures, while a radius of 1 and 2 produced qualitatively different networks. This was the basis for setting the WORDij default radius to three. Word embedding (Mikolov et al., 2013), like WORDij (Danowski, 1993a, 2013), also uses a five-word sliding window method. However, it treats word co-occurrences using a distance-based vector rather than a network model.

## Cascaded Semantic Fractionation

Cascaded Semantic Fractionation (CSF) stands out in semantic network analysis due to its multi-layered approach, contrasting with the standard network analysis that typically conducts a singular, surface-level examination. CSF delves deeper, iteratively exploring layers of semantic groups. This method increases granularity, systematically filtering out irrelevant links to focus more precisely on a specific semantic group representing a domain. Traditional network analysis generally adopts a single-level approach, lacking this iterative depth.

In essence, while conventional network analysis forms a horizontal two-dimensional structure, CSF creates a vertical articulation of the network to pinpoint a functional apex. The process begins with community detection to identify word groups, then hones in on the group containing initial seed terms. A new seed group is formed from these terms, and a network is built solely from these words. Community detection is applied again, and this fractionation process continues until it narrows down to a single, coherent community.

**Figure 1:**
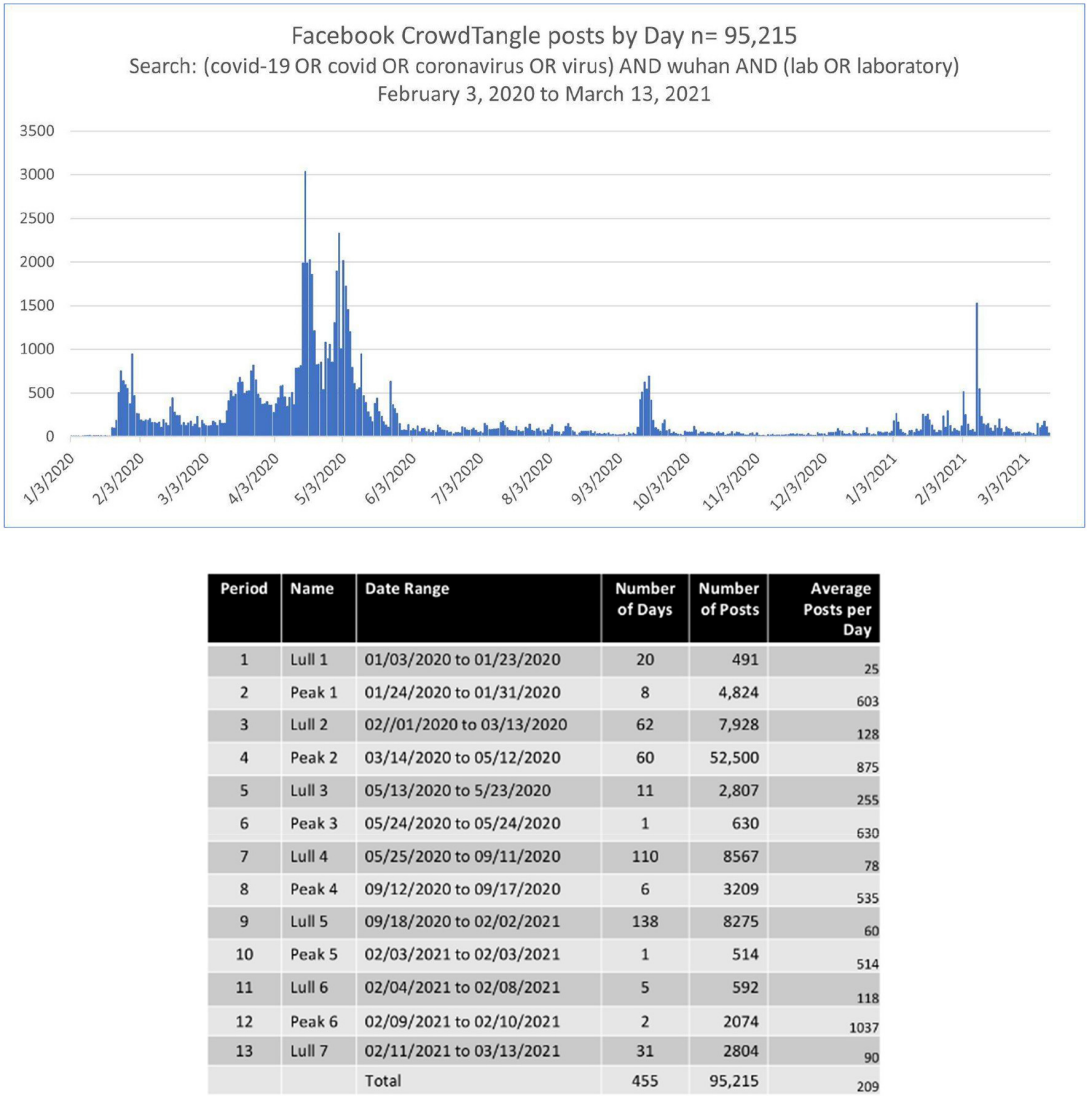
Wuhan lab posts by day.

Social media have two main categories of topics: stable and temporal (Yin et al., 2013). Temporal topics arise in bursts of posts over time (see Figure 1). Semantic network analysis of social media can take the bursty nature of topic formation and change into account in locating content domains of interest and pruning irrelevant content across time. The Cascaded Semantic Fractionation process steps are as follows:

1. Search the social media content to extract all content containing the search term seed.
2. Segment the corpus into days.
3. Identify burst periods. Examine the change in the number of posts from the run of prior days to the current day and determine the number of posts that marked the beginning of the burst. In the application example, we found an increase or decrease of 500 posts marked the beginning and end of a burst.
4. Aggregate text across bursts. This enables identifying text associated with increased posting.
5. Preprocess the data, removing stop words and punctuation and lowercase the text. We do not stem because it reduces semantic clarity. Even plural and singular forms of words have significantly different semantic networks (Danowski, 1993b).
6. Run semantic network analysis to generate word bigrams and their co-occurrence counts by passing a sliding window through the text.
7. Conduct a node-centric network extraction, capturing word pairs within two-link steps of the seed. The first step is the link between the seed and other words, while the second step is the links these words have with different words. Two-step links are significant in social network research, while there is mostly decay at three and higher-step links (Leskovec et al., 2007; Fowler and Christakis, 2008).
8. Perform community identification to fractionate the content into groups. Two equivalent methods are the Clauset et al. (2004) and Louvain algorithms, where the latter is a faster implementation of the same community detection based on modularity.
9. Locate the group that contains the seed term and use the terms in the group as an *include list* to map the network within the group.
10. Run community detection on this reduced network and locate the seed group.
11. Iterate steps 9 and 10 until each of the groups identified deals with aspects of the seed, and there is little cross-domain content across these groups.

The CSF process is similar to a binary search where, given an array of items, one divides them into the half that contains the target and the one that cannot, repeating this process until reaching the target. The difference is in the number of cuts, where binary search only creates two at each step, while CSF creates several groups, typically 3–4 major ones at each stage.

## Semantic network changes

The analysis of change over time in semantic networks has been approached differently. One approach is time-series analysis. It examines semantic networks over equal time intervals. For example, Danowski and Edison-Swift (1985) analyzed an organization's email over 12 months and examined the effects of a crisis. Similarly, studying change over time to compare semantic networks before and after intervention as a naturalistic field experiment, Danowski (2008) tested a hypothesis about semantic priming vs. framing in news coverage.

A continuous external variable can be aligned rather than events as the basis for examining change in semantic networks. A study of presidential cabinet network centrality and presidential job approval over time (Danowski and Cepela, 2010b) mined the social networks among the cabinets of President Reagan through G.W. Bush based on the members' co-occurrence in news stories. Each administration's data was sliced into time intervals corresponding to Gallup presidential approval polls to analyze the changes associated with them.

Danowski (2012a) illustrated a different time-based analysis in studying Muslim nations' networks associated with Jihad in web pages, broadcasts, newspapers, and other content. Unlike most semantic network analyses, nodes were time segments, not words. The link strengths were similarity scores of time nodes across word pairs. Analyzing sentiment changes over time, Danowski (2012b) collected and analyzed 5 years of documents mentioning the Taleban from Afghani and Pakistani sources and U.S. messages broadcast by Radio Free Europe/Radio Liberty (RFE/RL) connected with Afghanistan over the same 5-year period. Based on Fredrickson and Losada (2005), positivity ratios in each time slice during this period showed that Taleban content was generally evident of flourishing, while RFE/RL was consistently languishing.

In another approach to change in semantic networks over time, Danowski (2012c) analyzed change over time in Facebook's publics using an include list and mapping the publics over 12 months. In each time slice, networks were mapped among key publics, measuring the centrality of each from one time period to the next.

Other approaches to examining change over time in semantic networks are vector space modes such as Latent Semantic Analysis (LSA; Deerwester et al., 1990) and Word2Vec (Mikolov et al., 2013). Changes in the position of word vectors over time can reveal semantic shifts.

Topic Modeling uses methods like Latent Dirichlet Allocation (LDA; Jelodar et al., 2019) to identify topics in a corpus and how these topics evolve. This can reveal changes in the thematic structures of semantic networks by comparing changes in topics and their composition. Abuhay et al. (2021) used topic modeling to analyze the evolution of scientific research topics overtime on a corpus of computational science papers spanning 17 years.

The present research compares pairs of semantic network results to identify statistically significant changes in word pair proportions. When studying how a semantic network changes over time, some elements drop out, others are added, and the link strengths change. A suitable method is to compare the word pairs for the two points in time, identify significant differences, and then do community detection to interpret the changing meanings.

These steps analyze semantic network changes:

1. Compare the relative frequencies of word pairs for each consecutive pair of periods, i.e., period one vs. period two, period two vs. period three, etc., to identify word pairs that significantly increased or decreased. A *Z*-test for proportions reveals the significant changes in the composition of the semantic domain.
2. Run community detection on the significantly different bigrams for each period-to-period comparison.
3. Interpret the changes in bigrams.
4. Assess the over-time pattern pairs of periods for evidence of domain morphing.
5. Analyze the theoretical implications of the findings.

## Inter-media agenda setting

Inter-media agenda setting is a fruitful study area in media and communication research. The research investigates how different media entities influence each other's news agendas. Vliegenthart and Walgrave (2008) analyzed how nine news media in Belgium covered 25 issues over 8 years. They found that the impact of inter-media agenda-setting varies depending on factors like the time lag between coverages, the type of medium, language or institutional barriers, and the nature of the issue. Further studies in this field include those by Vonbun et al. (2016), Harder et al. (2017), and Nygaard (2020), which also explore inter-media agenda setting.

Su and Borah (2019) offer insights into the relationship between social media and traditional news outlets. Their study shows that Twitter significantly influences newspaper coverage during breaking news situations. In contrast, newspapers tend to guide Twitter's content during periods of ongoing discussion when there is no breaking news. This highlights the varying influence between different media types depending on the news context. Our research examines inter-media agenda-setting regarding the Wuhan lab and the coronavirus, considering the effects of Facebook posts on Fox News content.

## Application

### Data

In March 2020, Facebook launched its new CrowdTangle search interface to extract public posts and group pages over its history. In experimenting with the tool in the early pandemic, we noted that the prevailing argument for the emergence of COVID-19 in the mainstream media was a zoonotic animal-to-human transfer from a wet market. An alternative counter-argument emerged in social media that the virus escaped from the Wuhan Institute of Virology laboratory, where it was being weaponized. We used the lab-origin example to develop the methods reported here.

The CrowdTangle search tool includes access to historical records of public Facebook posts and group pages since the beginning of the social media. We used the search terms:

“(COVID-19 OR coronavirus OR virus) AND Wuhan AND (lab OR laboratory)” from November 1, 2019, to March 13, 2021, to obtain each post's metadata, resulting in a CSV file of 147.9 MB. Records for each post (*n* = 95,215) include the page or group name, user name, type of link, various interactions such as likes and shares, views, and the post text, as well as the descriptions of the links, photos, and videos, included in the semantic network analysis.

Figure 1 shows the distribution of posts from February 3, 2020, to March 13, 2021.

We plotted posts over time to determine the burst periods, identifying six. We grouped the posts for the six periods and analyzed their texts with semantic network analysis using the following steps:

1. Extract the relevant corpus by searching the CrowdTangle Facebook database.
2. Graph the number of posts by day.
3. Identify the surges of posts and extract text for each, forming an aggregated file.
4. Run the surge posts network in WORDij^2^ to identify and map the overall semantic network. We set the minimum frequency threshold to 100, given the overall volume.
5. Run a NodeTric conversion centered on “lab” with the number of steps = 2. This utility in WORDij enables extracting the links from 1 to n steps away from a seed term. Two is the preferred number of link steps because there is a steep decay after this.
6. Run community detection on the extracted seed-centric network and locate the group containing the seed.
7. Build an include list by selecting key terms from the group word list, and mapping the network of terms.
8. Identify the subgroups forming the word domain.
9. Take the words as an include group and run it against the aggregate network sliced into daily intervals to track changes.

We conducted a semantic network analysis by aggregating posts during identified bursts. Our focus was on word pairs occurring more than 100 times. We centered our node-centric analysis on the term “lab” due to its association with the Wuhan laboratory and the discourse regarding the origin of COVID-19 and because lab was the most frequently associated term with the posts on the origin of the coronavirus. The non-abbreviated “laboratory” was not as frequently used. Nevertheless, the node-centric network extraction captured this term, and the others linked at least 100 times. This method effectively maps the broader semantic networks in which discussions about the Wuhan lab are situated, providing insights into the related discourse domains.

**Figure 2:**
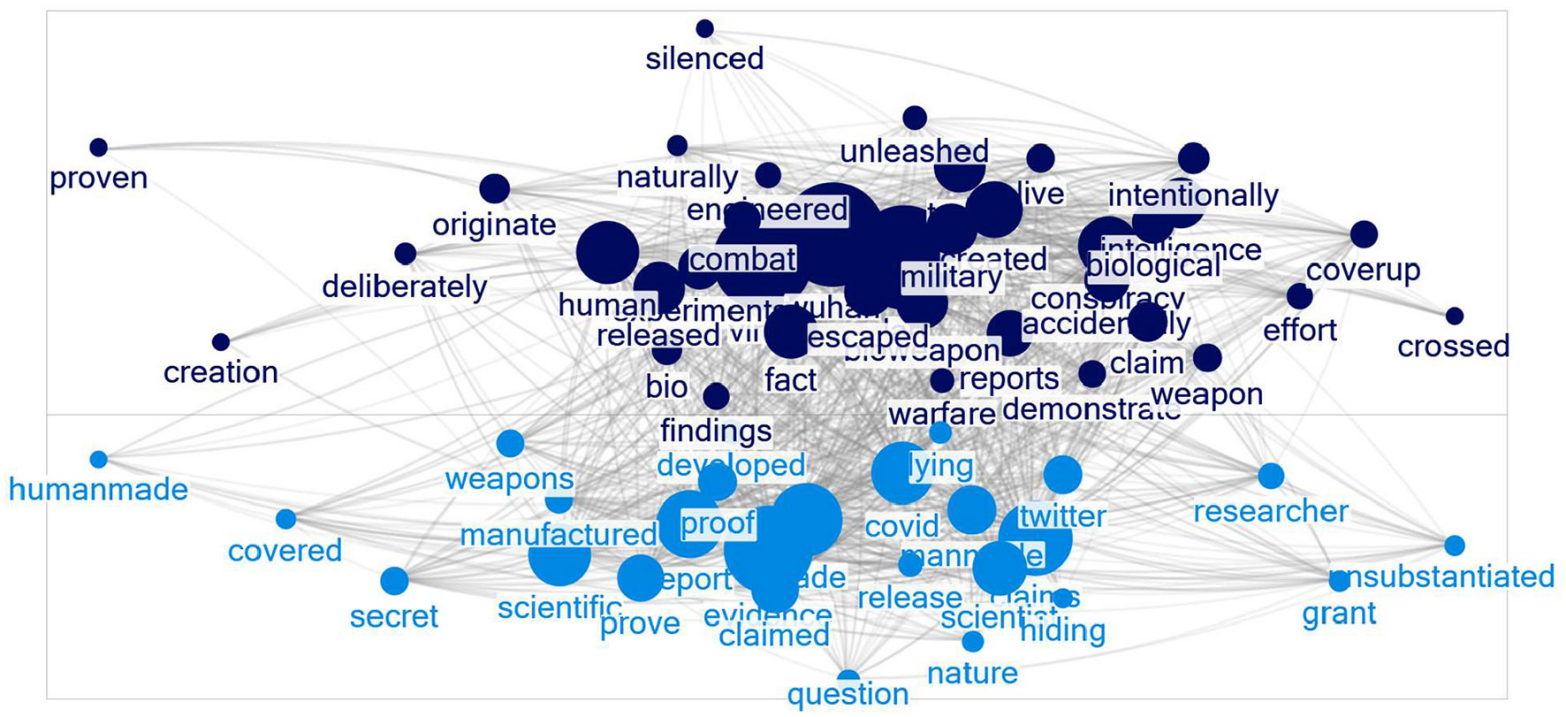
The Wuhan lab semantic domain.

This procedure produced a group of 526 words. We then identified the group that contained “lab” and created an include list with the group's words, resulting in 245 words. We reviewed each word on the list and selected words relevant to the Wuhan lab origin narrative, numbering 67, shown in Figure 2. These words formed a final include list we ran against each period's posts to see how the semantic domain changed over time.

We identified three groups in the network using the Caluset-Newman-Moore community detection method in NodeXL.^3^ The Clauset-Newman-Moore community detection algorithm (Clauset et al., 2004) identifies communities by finding the node cluster that produces the largest increase in modularity or the relative density of edges within communities to those outside communities. Other faster clustering algorithms, such as the Louvain method (Blondel et al., 2008), produce equivalent results. Our research utilized the Clauset-Newman-Moore algorithm because it is available in NodeXL, which we used to generate the networks.

Figure 2 shows the network of terms co-occurring 100 times or more across all bursts. The first group contains the main terms in the narrative that the coronavirus was human made and intentionally released. The second group focuses on the military bioweapon aspects.

### Change over time

With the six bursts of posts, it is possible to observe the change from one burst to the next by doing a series of *Z*-tests to observe which word pairs became more frequent, less frequent or showed no significant change. Due to space constraints, we do not report each pair-wise comparison of the six bursts. Instead, we compare the first burst to the last to see the overall change.

### Burst 1: January 24–31, 2020

The initial elements of the Wuhan lab domain emerged on January 24 to 31, 2020. The biological warfare element of the narrative is prominent, as seen in Table 1. It shows which word pairs were significantly more likely in Burst 1 compared to the final Burst 6.

**Table 1:** Top 20 word pairs significantly lower relative frequency in burst 6 vs. burst 1.

**Word pair**		**B1 FRQ**	**B6 FRQ**	**B1 PCT**	**B6 PCT**	***Z*-test**
Wuhan	Biological	2,340	7	0.058518	0.000487	29.34847
Biological	Warfare	2,234	4	0.055867	0.000278	28.75983
Biological	Weapons	1,788	7	0.044713	0.000487	25.44142
Wuhan	Weapons	1,068	0	0.026708	0	19.78189
Warfare	Wuhan	1,035	0	0.025883	0	19.46785
Biological	Wuhan	963	8	0.024082	0.000557	18.25753
Weapons	Biological	798	0	0.019956	0	17.05635
Virus	Biological	752	6	0.018806	0.000418	16.11946
Weapons	Warfare	665	0	0.01663	0	15.55095
Warfare	Virus	565	0	0.014129	0	14.32076
Biological	Virus	560	0	0.014004	0	14.25659
Virus	Weapons	524	3	0.013104	0.000209	13.5281
Intelligence	Biological	448	0	0.011203	0	12.73823
Military	Intelligence	402	0	0.010053	0	12.06141
Warfare	Biological	354	0	0.008853	0	11.31341
Military	Biological	349	0	0.008728	0	11.23271
Weapons	Wuhan	323	0	0.008077	0	10.8036
Nature	Wuhan	277	0	0.006927	0	10.00051
Lab	Biological	284	3	0.007102	0.000209	9.777704
Lab	Weapons	255	0	0.006377	0	9.593214

B1 PCT and B2 PCT are the word pair frequencies divided by the total number of word pairs in each burst, yielding proportions or percentages of the time a word pair occurs in the text segments.

### Burst 6: February 9, 2021 to February 10, 2021

Table 2 contains the word pairs that are significantly more likely in Burst 6 than in Burst 1. The focus is on the findings of the WHO report about the origins of COVID-19.

**Table 2:** Top 20 word pairs significantly higher relative frequency in burst 6 vs. burst 1.

**Word pair**		**B1 FRQ**	**B6 FRQ**	**B1 PCT**	**B6 PCT**	***Z*-test**
COVID	Wuhan	932	440	0.002092	0.030632	−61.725
COVID	Lab	750	308	0.001684	0.021442	−48.647
Lab	COVID	461	215	0.001035	0.014968	−42.897
Claim	COVID	19	75	0.000043	0.005221	−42.731
Wuhan	COVID	1,080	299	0.002425	0.020816	−39.675
Lab	Findings	49	83	0.00011	0.005778	−39.470
Findings	Virus	148	117	0.000332	0.008145	−38.403
Findings	Wuhan	225	125	0.000505	0.008702	−35.062
Lab	Wuhan	23,534	1,686	0.052832	0.117377	−33.440
Findings	Lab	66	66	0.000148	0.004595	−30.963
Findings	Human	4	34	0.000009	0.002367	−30.599
Virus	Lab	12,243	1,016	0.027485	0.070732	−30.486
COVID	Question	0	27	0	0.00188	−28.937
Wuhan	Findings	216	94	0.000485	0.006544	−27.537
Lab	Virus	10,518	812	0.023612	0.05653	−25.048
Wuhan	Lab	35,787	1,945	0.08034	0.135408	−23.669
COVID	Findings	3	20	0.000007	0.001392	−23.112
COVID	Originate	5	20	0.000011	0.001392	−22.096
Human	Wuhan	755	139	0.001695	0.009677	−21.375
Human	Lab	301	80	0.000676	0.005569	−20.063

B refers to Burst.

**Exemplary quotes**

- “Two facilities in Wuhan are linked to covert Chinese biological weapons programs. Did the deadly coronavirus escape from a BW weapons lab?”
- “A leading US Intelligence advisor has taken a firm stance on the origins of COVID-19, insisting that the virus came from a biological weapons lab in Wuhan.”
- “Obama and Fauci caused this bio-weapon virus to exist because they ILLEGALLY sent 3.7 million of our taxpayer dollars to Wuhan Lab in China.”
- “Is the deadly coronavirus from Wuhan, China a biological weapon, escaped from a Chinese lab, to be used as plague warfare?!”
- “A leading Italian Catholic historian says that there is more and more evidence that COVID-19 was not only manufactured in a Chinese laboratory but it was released by the communists in an act of “biological warfare” as part of their ‘program for the future.”'

The Burst 6 results show that the top 20 word pairs that occurred more frequently than in Burst 1 were about the findings of the WHO team that visited the lab to investigate. Exemplary quotes are:

- “WHO finds no evidence of corona virus in Wuhan lab.”
- “WHO Scientist Says No Evidence of COVID-19 Leak From Wuhan Lab Clean Chit To China.”
- “The WHO mission to China to uncover the origins of the coronavirus has failed to identify the source of the pandemic but the team on Tuesday ruled out the Wuhan lab-leak theory propagated by Donald Trump.”
- “Novel coronavirus is unlikely to have leaked from China's Wuhan lab, a World Health Organization expert said. WHO food safety and animal diseases expert Peter Ben Embarek made the assessment in a summation of a WHO team's investigation into the possible origins of the coronavirus in the central Chin…”

In summary, the over-time analysis shows the emergence of the Wuhan lab semantic domain, creating the conspiracy theory that the coronavirus was intentionally released as a bioweapon, the fading of that narrative, and the revision pointing to a possible accidental escape. At this stage, the domain is sufficiently cleansed of the intentional release narrative as it merges with the mainstream media narrative.

### Cable news coverage of “Wuhan AND lab AND COVID-19”

The inter-media agenda-setting analysis between social and mainstream media aimed to identify the venues with the most extensive viewership. Ratings showed Fox News, CNN, and MSNBC to have the most significant market shares (Schneider, 2020). Note that these channels' ideological orientation was rated in the Gallup/Knight (2020) study on media trust and democracy. MSNBC was rated as left at 1.0, CNN as left-center at 1.25, and Fox News as conservative at 4.75.

We searched the GDELT Television News Explorer.^4^ Fox News had the most domain coverage, with a relative air time scale value of 1.4. In contrast, CNN had 0.75, and MSNBC had 0.49. A question is how the two media interact concerning what was initially considered misinformation. There are four possible relationships between social media and mainstream media coverage of the semantic domain: social media may lead the mainstream coverage, the latter may reflect it, or refract it as it modifies the narrative, and it may lag the changes in social media.

### Relationship between Fox News and Facebook posts

**Figure 3:**
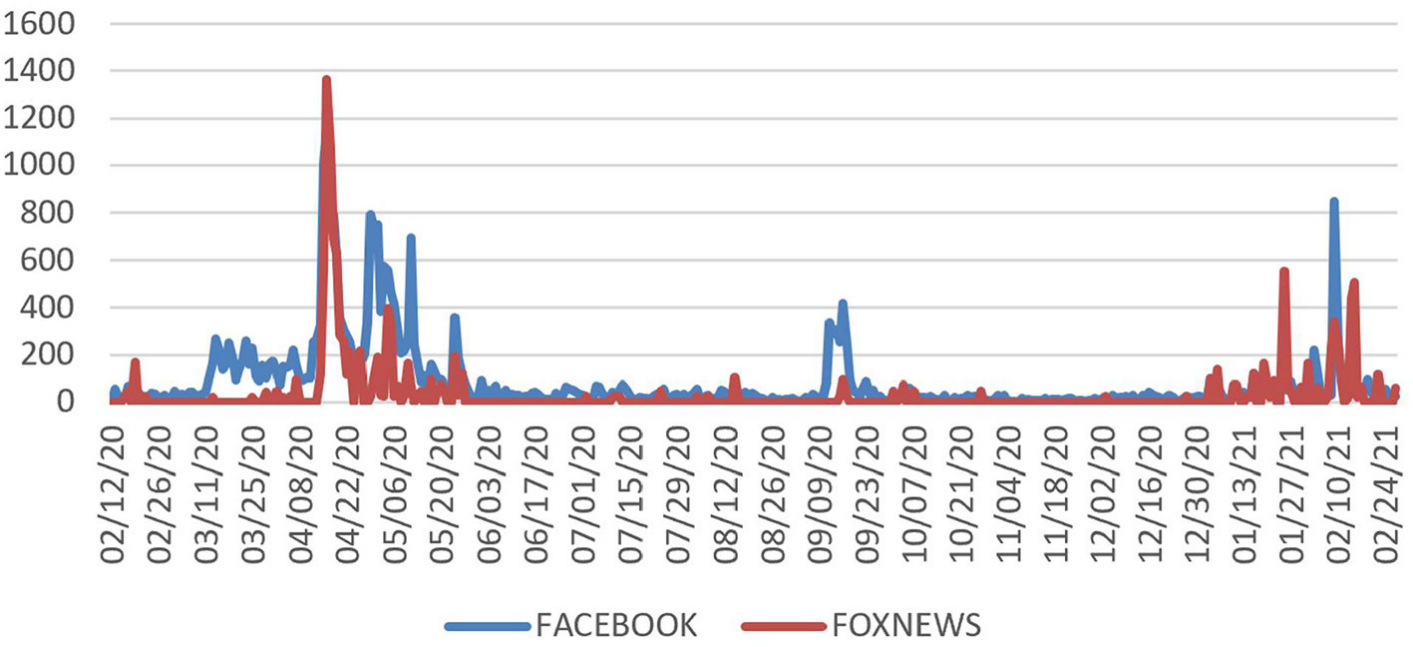
Fox News and Facebook posts in the main surge: 2/12/20–5/28/20.

Figure 3 shows the alignment of the curves for Facebook and Fox News during the main burst. Fox News coverage has the strongest relationship at the zero-lag point with *r* = 0.33 and a significant 1-day lag with *r* = 0.24.

### Cable news coverage of “Wuhan lab”

In addition to analyzing posts on Facebook, we examined the coverage of “Wuhan lab and COVID-19” on cable news channels with GDELT data for the three major domestic cable news channels. The spikes for coverage parallel those for Facebook posts. Among the domestic outlets, Fox News has most of the coverage. Links to its content on Facebook are among the most widely posted. The results show that Fox News coverage dovetailed with the Facebook posts about the Wuhan lab and was instrumental in the diffusion of the Wuhan lab semantic domain.

## Discussion

This research illustrated the Cascaded Semantic Fractionation method to extract the groups of words related to a seed from a large corpus of social media posts. Community detection and start-word include lists that enable digging below the surface-level network to prune cross-domain linkages iteratively and arrive at a local network structure that maps the domain of interest.

Polysemy and a high degree centrality draw multiple domains into large groups of words identified through community detection. The cross-domain linkages complicate analysis and interpretation. Cascaded Semantic Fractionation (CSF) iteratively removes the intergroup links from a domain using start-word include lists of intragroup nodes and repeats community detection on the links until the domain of interest has been clarified.

The first step was to create a daily series of the number of posts retrieved and to identify bursts where more than 500 posts were made; a number arrived at by examining the data. An increase of at least 500 posts marked the beginnings and ends of the bursts in these data. This burst-sensitive approach filters out the stable baseline content that functions as the constant of the trend. We analyzed the text from these post surges for one and two-step links from the seed term and ran community detection on these bigrams to map domains. This burst semantic network analysis method increases the likelihood that the most significant content will be included in the CSF. When the change over time is of interest, there is little value in analyzing the long stretches of low activity. This initial burst-based step in processing the posts reduced the extraneous content introduced at a low-frequency level.

Moreover, we also demonstrated how, when the change over time is of interest, successive post bursts can be tested for significant differences in word pairs, then analyzed with community detection to aid interpretation. We illustrated an over-time analysis of the daily series of posts. Substantively, across the year-long study period, the methods revealed the development of what was initially a conspiracy theory to become a plausible explanation for the search for the origin of COVID-19, a possibility that increases in likelihood in the 2 subsequent years that have passed.

On March 1, 2023, CNN reported:

FBI Director Christopher Wray on Tuesday acknowledged that the bureau believes the COVID-19 pandemic was likely the result of a lab accident in Wuhan, China. In his first public comments on the FBI's investigation into the virus' origins during an interview with Fox News, Wray said that “the FBI has for quite some time now assessed that the origins of the pandemic are most likely a potential lab incident in Wuhan.”

This research developed the Cascaded Semantic Fractionation (CSF) method, an iterative zoom-in through layers of semantic groups identified through a sequence of community detections, to identify a semantic domain of interest in social media. Additionally, a procedure for identifying changes in semantic networks over time was introduced. This involved comparing networks for significantly different word pairs and then running a community detection to reveal how meaning had shifted.

In this effort, we introduced tools tailored to link several utilities for semantic network analysis: WORD'j's WordLink for generating bigrams and its include list functionality, NodeTric for extracting links two steps from the seed term, and NodeXl's community detection and graphing functions.

Over time, the conspiracy associated with the intentional release of the COVID bioweapon under development at the Wuhan Institute of Virology dissipates until, eventually, the mainstream media considers both the zoonotic and lab leak narratives as plausible, creating an open question about the origin of COVID. The domain dissolves as it reaches the mainstream media surface, and both narratives are considered plausible and the subject of further investigation by intelligence and scientific communities.

The findings have several theoretical implications. The emergence and morphing of the semantic domain fit ((Hegel, 1807)) dialectic theory (cf. Forester, 1993; Zizek and Žižek, 2012) as a discursive process in which there is a dominant thesis, an antithesis challenge, and a synthesis of the two into a new thesis. In the case of COVID-19 origin, after ~1 year of discourse, beginning with the two opposing narratives (Calisher et al., 2020), the issue was characterized as an open question (Macias and Mendez, 2021) in the mainstream media with the lab origin gaining support (Gale, 2021).

The COVID origin case shows that the contemporary dialectical process involves the intermedia agenda-setting between the social and mainstream media narratives. After shedding conspiracy theory aspects, the social media narrative transforms the mainstream media narrative as it merges with it. What was initially labeled as misinformation based on rumor and conspiracy theories ascends to information status. The interactivity and engagement of social media appear to enhance the dialectical process. Before the development of social media, mainstream media were considered agenda-setting agents influencing public perceptions of the importance of issues (McCombs and Shaw, 1972) and how they are framed (De Vreese, 2005; Tewksbury and Scheufele, 2009) in a one-way process from traditional media to public attitudes. In contrast, social media creates a discursive process that interacts with the mainstream media's traditional agenda-setting to change the agenda over time (Feezell, 2018).

### Contributions

The novel Cascaded Semantic Fractionation method addresses the challenge of polysemy and high-degree centrality in semantic network analysis. CSF iteratively prunes intergroup links from a domain, using start-word include lists of intragroup nodes, and repeats community detection until the domain of interest is clearly defined. This approach helps isolate specific semantic domains from the complex web of social media texts.

The Burst Semantic Network Analysis method introduces a burst-sensitive approach to data collection. It involves creating a daily series of the number of posts and identifying significant surges in posting activity (bursts). This approach filters out stable baseline content, allowing for a focus on the most significant content for analysis. It is particularly effective in reducing the noise of low-frequency content in large datasets.

The research integrates several analytical tools for semantic network analysis. This includes using WORD'j's WordLink for generating bigrams, NodeTric for extracting links two steps from the seed term, Z Utilities's *Z*-test Pairs for assessing change over time, and NodeXl for community detection and graphing. This integration showcases a holistic approach to semantic network analysis, leveraging the strengths of various existing tools.

The methodology also includes a procedure for identifying changes in semantic networks over time. This is done by comparing networks for significantly different word pairs. This methodology aspect is crucial for understanding how meanings and narratives evolve in social media.

These methodological contributions significantly enhance the ability to analyze and interpret complex semantic networks in social media. They provide a more nuanced understanding of how narratives and discourse develop and change in the digital age, especially in the context of large and noisy datasets.

## Conclusion

In conclusion, as demonstrated in this study, the Cascaded Semantic Fractionation (CSF) method represents a significant advancement in analyzing semantic constructs in social media. By applying this method to the contentious discourse surrounding the origin of COVID-19, we have illustrated its effectiveness in dissecting complex social media narratives. The CSF method's ability to iteratively drill down through layers of semantic groups, identified via a sequence of community detections, provides a nuanced understanding of how misinformation and different narratives evolve on social media platforms.

Our findings highlight social media discussions' dynamic and multifaceted nature, showing how various groups shape a narrative over time. As mapped by the CSF analysis, the evolution of the COVID-19 origin controversy underscores the importance of timely and accurate information dissemination in managing public health crises.

Moreover, the CSF approach offers a valuable tool for researchers, policymakers, and communicators in identifying and addressing the spread of misinformation. By understanding the trajectory and transformation of narratives, stakeholders can develop more effective strategies for public communication and policymaking, especially in uncertain scenarios and rapidly evolving information.

Future research should aim to apply the CSF method to other complex and evolving topics in social media, further refining the technique and exploring its broader applications. The potential of CSF in enhancing our comprehension of semantic networks in digital communication promises a deeper insight into the intricacies of social media narratives and their impact on public discourse and opinion.

## Data availability statement

The raw data supporting the conclusions of this article will be made available by the authors, without undue reservation.

## Author contributions

JD designed the study, collected the data, analyzed the data, and wrote the paper. KR collaborated throughout. BY had the initial idea for the research and assisted in data analysis and editing. All authors contributed to the article and approved the submitted version.

## Footnotes

^1^For NEGative EntrOPY, a name Danowski suggested when they developed the network analysis program while students in the Department of Communication at Michigan State.

^2^ https://wordij.net

^3^ https://www.smrfoundation.org/nodexl/

^4^ https://api.gdeltproject.org/api/v2/summary/summary?d=iatv
